# La chirurgie coronaire sous circulation extra-corporelle chez le patient diabétique

**DOI:** 10.11604/pamj.2014.17.199.2379

**Published:** 2014-03-13

**Authors:** Younes Moutakiallah, Khadija Benzaghmout, Mahdi Aithoussa, Nourreddine Atmani, Brahim Amahzoune, Abdedaim Hatim, Mohamed Drissi, Aatif Benyass, Youssef ElBekkali, Abdelatif Boulahya

**Affiliations:** 1 Service de Chirurgie Cardiaque, Hôpital militaire d'instruction Mohammed V Rabat, Maroc; 2 Service de Cardiologie, Hôpital militaire d'instruction Mohammed V Rabat, Maroc; 3 Réanimation de Chirurgie Cardiovasculaire, Faculté de Médecine et de pharmacie. Université Mohammed V, Rabat, Maroc

**Keywords:** Pontage aorto-coronaire, diabète, morbi-mortalité, CABG, diabetes, morbi-mortality

## Abstract

Nous rapportons les résultats de la chirurgie coronaire chez une population de coronariens diabétiques opérés sous circulation extra-corporelle dans le service de chirurgie cardiaque de l'hôpital militaire d'instruction Mohammed V de Rabat. C'est une étude rétrospective menée entre Janvier 2008 et Février 2012 (4 ans), portant sur 103 patients diabétiques consécutifs opérés pour pontage coronaire. L’âge moyen des patients était de 61±8,7ans (37-82ans) avec un sexe ratio (H/F) de 3,9. Tous les patients diabétiques de type 2 et sous traitement anti-diabétique ont été inclus dans cette étude. La sténose du tronc commun gauche était présente chez 26,2% des patients et 53,4% étaient tritronculaires. Quatre-vingt patients (78,6%) étaient insulino-nécessitant, l'Euro-score moyen était de 1,63±1% et le nombre moyen de pontage de 2,3±0,7. Les durées moyennes de la circulation extra-corporelle et du clampage aortique étaient respectivement de 134,4 ± 42 min et 76 ± 28 min. La mortalité hospitalière était de 2 décès (1,9%), les durées moyennes de ventilation artificielle, du séjour en réanimation et du séjour postopératoire étaient respectivement de 7h (5-16h), 48h (42-52h) et 15,6 ± 8,6 jours. Les complications postopératoires étaient l'infarctus du myocarde, l'infection de paroi, la médiastinite et le bas débit cardiaque chez respectivement 1,9%, 10,7%, 3,9% et 1,9% des patients. Il ressort de notre étude, que les facteurs prédictifs d'infection post opératoire étaient la durée de ventilation artificielle (p = 0,002), la durée de la circulation extra-corporelle (p < 0,001) en plus du tabac (p = 0,004) et de l'obésité (p = 0,005). Les patients ont été contactés par téléphone ou lors de la consultation régulière de contrôle. Le taux de suivi a atteint 92,1% et la survie à 2 ans était de 98,9% des patients contrôlés avec une mortalité tardive de 1% avec un décès suite à un accident vasculaire cérébral étendu. Le contrôle a montré un cas de ré-hospitalisation pour poussée d'insuffisance cardiaque mais aucun cas de récidive angineuse, d'infarctus du myocarde ou de besoin de revascularisation. La chirurgie coronaire chez le diabétique offre d'excellents résultats à court et à moyen terme au détriment d'une faible morbi-mortalité ce qui en fait le traitement de choix.

## Introduction

Le diabète est en constante progression dans le monde, on estime le nombre des diabétiques à 170 millions dont 90% de type II. Au Maroc, la prévalence du diabète est de 6,6% ce qui en constitue un problème de santé publique [1]. La progression galopante du diabète et de la coronaropathie dans la population marocaine a largement influencé notre activité au sein du service de chirurgie cardiaque de l'hôpital militaire d'instruction Mohammed V (HMIMV) de Rabat. Une importante proportion de patients diabétiques est de plus en plus proposée à une chirurgie coronaire. Le pontage aorto-coronaire (PAC) est le traitement de référence chez le diabétique. Cependant, les comorbidités et les atteintes dégénératives rendent la gestion péri-opératoire plus délicate dont la clé de voute est le strict contrôle glycémique péri-opératoire et la revascularisation « tout-artériel » la plus complète possible. Le but de ce travail est de présenter les résultats à court et à moyen terme d'une série de 103 patients diabétiques consécutifs opérés par PAC sous circulation extra-corporelle (CEC) sur une période de 4 ans étalée de Janvier 2008 à Février 2012. Nous comparerons ces résultats avec ceux de la littérature et déterminerons les facteurs prédictifs de morbi- mortalité.

## Méthodes

Sur étude des dossiers médicaux des patients, nous avons rempli des fiches individuelles contenant les informations cliniques et péri-opératoires des patients. Le Diabète est défini par une glycémie à jeun (GAJ) ≥ 1,26g/l ou une glycémie ≥ 2g/l après 120 minutes d'une épreuve hyperglycémie provoqué par voie orale. Ont été inclus dans cette étude, tous les patients diabétiques de type 2 et nous avons exclu, les patients diabétiques sous régime seul sans traitement médical et les patients diabétiques de type 1.

Tous les patients ont été opérés par la même équipe et selon le même protocole opératoire. Les ADO et l'insuline ont été arrêtés au moins 48h avant la chirurgie et remplacés par un protocole d'insuline ordinaire (IO) en sous cutané (Tableau 1), adapté en fonction de la glycémie capillaire (GC) répété toutes les 4h. Le traitement médical anti-ischémique, y compris l'aspirine, a été maintenu chez tous les malades jusqu'au jour de l'intervention, exception faite pour les inhibiteurs de l'enzyme de conversion qui ont été relayé par l'amlodipine pendant au moins 48h et le clopidogrel qui est relayé par l'enoxaparine pendant une semaine sauf urgence. La préparation du patient débute la veille de la chirurgie par une prémédication à base d'Hydroxine 100 mg en 2 prises. L'induction anesthésique et l'entretien ont été faits par l'association Midazolam, Thiopental, Propofol et de Bromure de pancuronium. L'antibio-prophylaxie a été assurée par Céfuroxime à la dose de 1,5g à l'induction puis 750mg en IV toutes les 6h pendant 48h ou la Vancomycine 15mg/kg en une heure en cas d′allergie. Le ballon de contre-pulsion intra-aortique (BCPIA) a été inséré en cas d'instabilité hémodynamique ou en cas de sténose du tronc commun gauche (TCG) non protégée. Après sternotomie, les artères mammaires internes (AMI) commençant par l'artère mammaire interne gauche (AMIG) puis droite (AMID) ont été prélevées. La veine saphène interne (VSI) est prélevée au niveau des jambes par une incision unique. Dernièrement, la tendance dans le service est de prélever de plus en plus les deux AMI et de façon squelettisée.

**Tableau 1:** Algorithme du Protocole de l'insuline en fonction de la glycémie capillaire

Glycémie capillaire mg/dl	< 150	150 – 199	200 – 249	> 250
Insuline ordinaire (UI)	0	5	10	15

La CEC est installée entre une canule aortique et une canule veineuse atrio-cave après bolus d'héparine (300UI/Kg) pour atteindre un temps de coagulation activé supérieur à 400 secondes. La CEC est conduite en hémodilution totale avec hypothermie modérée à 32°C. La protection myocardique est assurée par une cardioplégie cristalloïde froide (saint thomas) injectée par la racine aortique en plus de la réfrigération péricardique. On procède par le pontage du réseau coronaire droite, circonflexe et enfin l'interventriculaire antérieure (IVA). En fin de procédure, la CEC est arrêtée. L'héparine est neutralisée par la protamine dose pour dose et la prévention du saignement a été assurée par l'injection de l'acide tranexamique à la dose de 15-30mg/Kg en 2 prises avant l'héparine et après protamine.

Le patient est transféré en réanimation pour complément de surveillance; 250mg d'aspirine est injectée en IV et l'héparine à la seringue auto-pulsée (SAP) (100UI/Kg/j) à la 6e h et la simvastatine est administrée le soir de l'intervention à la dose de 40mg/j; Les β bloquants ont été repris à J3 à base de carvédilol à la dose de 6,25mg/j en 2 prises. La glycémie a été étroitement surveillée au bloc par une glycémie capillaire (GC) horaire et correction par l'IO en IV; en réanimation l'IO est administrée à la SAP (40UI d'IO diluée dans 40cc de serum) avec ajustement de la vitesse de la SAP en fonction de la GC horaire. Dans le service, les patients sont remis au même protocole 1. Le traitement antidiabétique (ADO, insuline ou association) initial est repris 48h après.

L′analyse statistique a été réalisée avec le logiciel SPSS 17 (Chicago, Illinois, USA). Les variables quantitatives ont été exprimées en moyennes et écart-type ou en médianes et interquartile, et elles ont été comparées par le test de Student et le test non paramétrique U de Mann-Whitney. Les variables qualitatives ont été décrites par l'effectif et la proportion et ont été comparées par le test de Chi-2 ou le test des probabilités exactes de Fisher en fonction des conditions de validité. Les facteurs de risque potentiels avec P <0,20 en analyse univariée ont été inclus dans l′analyse multivariée, qui a été réalisée par régression logistique automatisée. La valeur P <0,05 était considérée comme seuil de significativité.

## Résultats

Définitions: La mortalité hospitalière est définit par tout décès survenant dans les 3O jours suivants la chirurgie ou durant l'hospitalisation. La morbidité a été définit par les événements cardio-vasculaires et cérébraux majeurs comme l'infarctus du myocarde (IDM), le bas débit cardiaque (BDC), l'accident vasculaire cérébral (AVC) et d'autre part la ventilation artificielle (VA) prolongée, l'insuffisance rénale (IR), la pneumopathie, l'infection de paroi et la médiastinite. L'IDM a été défini par une augmentation de la troponine 5 fois supérieure au 99e percentile au cours des 72 premières heures après la chirurgie et par l'apparition de nouvelles ondes Q pathologiques ou d'un nouveau bloc de branche gauche, ou bien par l'occlusion nouvelle d'une coronaire native ou d'un greffon documentée par une coronarographie, ou encore par la preuve d'une nouvelle perte de myocarde viable à l'examen d'imagerie. Le BDC a été défini sur des critères cliniques avec une pression artérielle systolique ≥ 80mmHg, une pression veineuse centrale < 10mmHg, une diurèse < à 0,5ml/Kg/h. La VA a été considérée comme pathologique quand elle dépasse 24h.

L’âge moyen des patients était de 61±8,7ans (37-82ans). L'angor était le signe révélateur le plus fréquent (54,4%). Les données préopératoires sont résumées dans leTableau 2. Sur le plan du bilan biologique, 39% des patients étaient bien équilibrés contre 61% qui étaient difficiles à équilibrer en se basant sur la GAJ (1,26-3,92g/l) et l'hémoglobine glyquée (HbA1c). On a noté une insuffisance rénale (IR) chez 5 patients. La CRP était positive chez 67% des patients avec une moyenne de 8,3±16,4mg/l (0,1-82,4mg/l). Concernant le traitement, 21,4% des patients étaient sous ADO, 69,9% sous insuline et 8,7% sous association ADO-insuline. L'association BASI (β bloquant, statine, antiagrégant plaquettaire et IEC) était prescrite chez 85% des patients et 10,6% étaient sous clopidogrel.

**Tableau 2:** Les données préopératoires de la population

Variable	N = 103	
	N	%
Âge (années)	61±8,7 (37–82)	
Sex-ratio (homme / femme)	3,9	
Nombre moyen de FDRCVx	1,9±1,1 (1–5)	
Nombre de FDRCVx ≥ 3	66	64,1
Tabagisme	57	55,3
HTA	47	45,6
Diabète insulino-nécessitant	81	78,6
Insuffisance rénale	5	4,9
IDM récent < 90 jours	16	15,5
Angioplastie	7	6,8
Artériopathie extra-cardiaque	6	5,8
BPCO	6	5,8
CCS 3-4	41	39,8
SCA	16	15,5
NYHA III-IV	6	5,8
IMC (Kg/m^2^)	25,9±4,5 (17,3-36)	
IMC ≥ 30 Kg/m^2^	10	9,7
Créatininémie (mg/l)	10,8±3,8 (5,7-81)	
RRS	103	100%
RCT	0,5±0,05 (0,36–0,7)	
FE (%)	54,6±12 (28 – 80)	
FE < 50%	20	19,4
FE ≤ 30%	2	1,9
Euroscore (%)	1,63±1 (0,5–5)	
Sténose du TCG	27	26,2
Monotronculaire	6	5,8
Bitronculaire	23	22,3
Tritronculaire	55	53,4
IVA proximale	93	90,3

**FDRCVx :** Facteur de Risque Cardiovasculaire ; **HTA :** Hypertension Artérielle **; IDM :** Infarctus du Myocarde ; **BPCO :** Broncho-Pneumopathie Chronique Obstructive ; **CCS :** Canadian Cardiovascular Society Classification ; **SCA :** Syndrome Coronarien Aigu ;**NYHA :** New York Heart Association ; **IMC :** Index de Masse Corporelle ; **RRS :** Rythme Régulier Sinusal ; **RCT :** Rapport Cardiothoracique ; **FE :** Fraction d'Ejection ; **TCG :** Tronc Commun Gauche ; **IVA :** Interventriculaire Antérieure.

La chirurgie était urgente (<24h) dans 9,7% des cas qui ont été opérés sous Clopidogrel. L'induction anesthésique a été faite sous BCPIA chez 7,8% des patients. Nous avons utilisé comme greffon l'AMIG, l'AMID et la VSI chez respectivement 97 (94,2%), 12 (11,6%) et 76 patients (73,8%). Le prélèvement mammaire a été fait de façon squelettisée chez 19,4% des patients et le montage artériel en Y a été réalisé dans 8,7% des cas. La sortie de CEC était facile dans 69% des cas et laborieuse dans 31% nécessitant une assistance par des drogues inotropes positives avec le recours au BCPIA chez un patient.

La Mortalité hospitalière, était de 1,9% avec deux décès par choc cardiogénique réfractaire à J4 et à J9. L'assistance par BCPIA a été utilisée dans 0,9% des cas et par les drogues inotropes dans 4,9% des cas. Les suites étaient simples dans 72,8% des cas, alors que 27,2% ont présenté des complications à savoir l'IDM postopératoire, le bas débit cardiaque, l'accident vasculaire cérébral (AVC), l'IR et la reprise chirurgicale (Tableau 3).

**Tableau 3:** Les données opératoires de la population

Variable	N =103	
	N	%
Chirurgie semi-urgente (24-72h)	10	9,7
Chirurgie urgente < 24h	10	9,7
BCPIA à l'induction	8	7,8
AMIG	97	94,2
2 AMI	12	11,6
Revascularisation « tout artériel »	24	23,3
VSI	76	(73,8%)
Nombre de PAC/patient	2,3±0,7 (1-4)	
Durée de CEC (mm)	134,4±42 (41-236)	
Durée du clampage aortique (mn)	76±28 (28-180)	
Durée de VA (h)	7 (5-16)*	(Extrêmes : 2-216)
VA ≥ 24h	10	9,7
Durée du séjour en réanimation (h)	48 (42-52)*	(Extrêmes : 24-672)
Durée du séjour postopératoire (j)	15,6±8,6 (4-51)	
Complications	75	72,8
IDM postopératoire	2	1,9
Bas débit cardiaque	2	1,9
AVC	1	0,9
Fibrillation atriale	2	1,9
Insuffisance rénale	2	1,9
Saignement total (cc)	821±536 (275-3000)	
Transfusion sanguine	21	20,4
Transfusion sanguine > 2 unités	4	3,9
Reprise chirurgicale	6	5,8
Infection de paroi	12	10,7
Médiastinite	4	3,9
Mortalité hospitalière	2	1,9

**BCPIA :** Ballon de Contre-Pulsion Intra-Aortique ; **AMIG :** Artère Mammaire Interne Gauche ; **AMI :** Artère Mammaire Interne ; **VSI :** Veine Saphène Interne ; **PAC :** Pontage Aorto-Coronaire ; **CEC :** Circulation Extra-Corporelle ; **VA :**Ventilation Artificielle ; **IDM :** Infarctus du Myocarde ; **AVC :** Accident vasculaire Cérébral.

* Médiane et écart interquartile

Le suivi a été fait soit par consultation des dossiers de suivi des patients (visites médicales des contrôles programmés) ou par contact téléphonique des patients. Sur les 101 patients survivants, 8 (7,9%) ont été perdu réalisant un taux de suivi de 92,1%. La survie à 2 ans était de 98,9% des patients contrôlés avec un décès suite à un AVC étendu. Le contrôle a montré un cas de ré-hospitalisation pour poussée d'insuffisance cardiaque mais aucun cas de récidive d'angor, d'IDM ou de besoin de geste de revascularisation.

## Discussion

Le diabète est connu pour être un puissant facteur de risque cardiovasculaire et la maladie coronaire chez le diabétique revêt certaines spécificités à savoir, le caractère diffus d'une part de la coronaropathie qui est sévère et d'autre part de l'athérosclérose qui est multifocale. Ainsi, chez le diabétique la coronaropathie est caractérisée par les atteintes tritronculaires, les sténoses du tronc commun gauche (TCG) et de l'IVA proximale. Cette atteinte tritronculaire a été présente 80,3% des patients de la série de Weintraub [2], et chez Yusuf, elle était présente chez plus de 40% des patients diabétiques contre seulement un quart des non diabétiques [3]; dans notre série l'atteinte tritronculaire a été retrouvée chez 53,4% de nos patients qui présentaient également 26,2% de cas de sténose du TCG et 90% de sténose proximale de l'IVA, ce même chiffre a été retrouvé dans l'essai MASS II [4].

La plupart des études qui ont comparé le PAC au traitement médical et à l'angioplastie (BARI 2D, CARDia, ARTS II et MASS II) [4–7] ont montré la supériorité du PAC concernant la mortalité opératoire, les événements cardiovasculaires majeurs, la récidive d'angor, la nécessité de geste de revascularisation ultérieure et la survie à court et à long terme. La mortalité hospitalière au cours du PAC sous CEC se situe généralement autours de 1,5-3% [8, 9] et entre 1 et 5% chez le diabétique [10, 11]. Dans notre étude, elle était de 1,9%.

Le contrôle glycémique péri-opératoire réduit la mortalité de 43% [12]. Une récente étude [13] a montré le bénéfice clair de maintenir une glycémie entre 110 et 140mg/dl en postopératoire immédiat d'une chirurgie cardiaque en adoptant un protocole d'infusion continue d'IO qui a été prouvé meilleur que le protocole d'ajustement par injection de bolus d'IO répété en fonction de la GC que ça soit pour le contrôle des chiffres glycémique que pour la prévention d'hypoglycémie qui était moins fréquente avec l'infusion continue d'IO. Dans notre série, nous avons mis comme GC cible entre 120 et 150mg/dl, 24,3% des patients avaient une GC moyenne dans la zone thérapeutique, alors que 48,5% des patients avaient une GC moyenne entre 150 et 200mg/dl alors que 27,9% des patients dépassaient 200mg/dl. Le taux d'IDM postopératoire était dans notre série de 1,9%, ce taux varie de 0,1 à plus de 10% selon les études, avec une moyenne de 2,4-3,4% [14] avec un impact sur la mortalité hospitalière qui passe de 3% dans le groupe sans élévation de la troponine à 7% dans le groupe où elle s′élève sans dépasser 1,5ng/ml et atteint 22% en cas d′IDM [15, 16].

Le risque de médiastinite est important chez le diabétique pouvant dépasser 10% [17] avec une mortalité élevée pouvant atteindre 14 à 40% [18]. Dans notre série, le taux de médiastinite est de 3,8%. Une méta-analyse [17] a concerné 140 études randomisées ayant traité la relation qui existe entre la médiastinite et le prélèvement des deux AMI. Elle a montré que le risque de médiastinite s’élève de 2,5 à 5 fois plus si les deux AMI ont été prélevées. Chez le ND, ce risque passe de 0,2-1,2% pour une seule AMI à 1,3-4,7% pour les deux AMI et peut dépasser 10% chez le diabétique. Ce risque baisse drastiquement pour atteindre 0,4-2,6% en cas de prélèvement squelettisé. Cette méta-analyse recommande fortement le prélèvement squelettisé des deux AMI même chez le diabétique ou plutôt surtout chez le diabétique qui en tire le plus de bénéfice. Dans notre expérience, les deux AMI ont été utilisées chez 11,6% des patients avec 8,7% de montage en Y et 23,3% de pontage mammaire exclusif; notre penchant actuel se fait vers l'utilisation des deux AMI squelettisées puisque le prélèvement squelettisé des AMI représente ces deux dernières années 60,8% des cas contre seulement 19,6% les deux premières années de l’étude et cette tendance se confirme plus la dernière année avec 76% de prélèvement squelettisé. Bien qu'elle soit moins grave que la médiastinite, l'infection de paroi est plus fréquente chez le diabétique avec un impact sur l’équilibre glycémique, le séjour hospitalier et le coût total d'hospitalisation [19, 20]. Dans notre série, elle était de 10,7% en accord avec certaines études [21, 22] et en désaccord avec d'autres [23]. Le diabète apparaît comme facteur prédictif indépendant d'infection de paroi après PAC, malgré les mesures préventives [21]. Les autres facteurs prédictifs sont l'hospitalisation préopératoire prolongée, l'obésité, le tabac, le sexe féminin, le nombre de PAC, l'usage des deux AMI et la durée de CEC [24]. Dans notre étude, les facteurs prédictifs d'infection étaient la durée de VA au-delà de 24h (p = 0,02), la durée de CEC dépassant 180min (p < 0,001), le tabagisme (p = 0,019) et l'obésité (p = 0,035). Outre les complications infectieuses, les complications neurologiques sont plus fréquentes chez le diabétique avec 24,3% versus 16,9% chez le non diabétique [25]. Dans notre série, nous comptons 0,9% d'AVC et 11,7% de troubles psychiques mineurs transitoires à type d'agitation ou hallucinations.

**Limites de l’étude**: Le caractère rétrospectif de notre série avec toutes les insuffisances rencontrées avec ce genre d’étude est surement un inconvénient. Egalement, le contrôle qui n'inclut pas une imagerie des artères coronaires (coronarographie ou coro-scanner) pour vérifier la perméabilité des anastomoses.

## Conclusion

Le diabète, maladie de plus en plus fréquente, est associé à une athérosclérose plus diffuse et évolutive chez le coronarien et sujette à des complications postopératoires. La rigueur de prise en charge aussi bien du diabète que de la coronaropathie est la seule garante d'une meilleure issue. Les résultats de la chirurgie ont connu une nette amélioration grâce à l'utilisation croissante des AMI, la revascularisation complète « tout artériel ».
